# Beneficiaries’ satisfaction with community-based health insurance services and associated factors in Ethiopia: a systematic review and meta-analysis

**DOI:** 10.1186/s12962-024-00541-4

**Published:** 2024-10-18

**Authors:** Ewunetie Mekashaw Bayked, Husien Nurahmed Toleha, Segenet Zewdie, Asnakew Molla Mekonen, Birhanu Demeke Workneh, Mesfin Haile Kahissay

**Affiliations:** 1 Department of Pharmacy, College of Medicine and Health Sciences (CMHS), Wollo University, Dessie, P.O. Box: 1145, Ethiopia; 2 Department of Pharmacy, College of Medicine and Health Science, Injibara University, Injibara, Ethiopia; 3 Department of Health Systems and Management, School of Public Health, College of Medicine and Health Sciences, Wollo University, Dessie, Ethiopia; 4 Department of Pharmaceutics and Social Pharmacy, School of Pharmacy, College of Health Sciences, Addis Ababa University, Addis Ababa, Ethiopia

**Keywords:** Beneficiaries, Satisfaction, Community-based health insurance, Factors, Ethiopia

## Abstract

**Background:**

The viability of community-based health insurance programs depends on beneficiary satisfaction, and healthcare systems evaluate performance through patient reports and ratings to ensure effectiveness and service quality. To our knowledge, Ethiopia lacks national pooled data on the satisfaction of community-based health insurance beneficiaries and related factors. As a result, this review aimed to evaluate the level of beneficiaries’ satisfaction with the scheme’s services and associated factors in Ethiopia.

**Methods:**

Database searches on Scopus, Hinari, PubMed, Google Scholar, and Semantic Scholar were conducted on September 1st, 2022. Thirteen studies were chosen for review from the search results. Checklists from the Joan Briggs Institute were used to evaluate the risk of bias for the included studies. The data were extracted using a 2019 Microsoft Excel spreadsheet and analyzed using Stata 17. The odds ratios at p-values less than 0.05 with a 95% confidence interval were used to evaluate the effect estimates.

**Results:**

The pooled satisfaction of beneficiaries with community-based health insurance was found to be 66.0% (95% CI = 57-76%) and was found to be influenced by socio-demographic, health service-related, the scheme’s related factors, and the beneficiaries’ knowledge of it. The beneficiary satisfaction levels were highest in the Amhara region, at 69.0% (95% CI = 59-79%), followed by Southern Nations Nationalities and Peoples' Region (SNNPR) at 67.0% (95% CI = 40-94%), Oromia at 63.0% (95% CI = 58-68%), and Addis Ababa at 53.0% (95% CI = 45-62%).

**Conclusion:**

Even though there was a moderate level of satisfaction, there are indications that the quality of health services and the coverage of the entire population lag behind, necessitating greater efforts to achieve universal health coverage.

**Supplementary Information:**

The online version contains supplementary material available at 10.1186/s12962-024-00541-4.

## Introduction

Ethiopia is the tenth-largest nation in Africa [1]. It is the most populous landlocked nation on the African continent. After Nigeria, it is the continent’s second-most populous nation. Its capital and largest city is Addis Ababa [2]. Beginning with the Alma-Ata Declaration on Primary Health Care (PHC) in 1978, it has taken a number of initiatives to attain universal PHC. The financing of its healthcare system comes from a variety of sources, including loans and donations from all over the world (46.8%), the government (16.5%), individual contributions (35.8%), and others (0.9%) [3].

Since 1993, various reforms in the health sector have been undertaken in Ethiopia [4–6] envisioned at the healthcare sector development (HSD) in the coming 20 years [6]. However, the health status of the country is among the worst not only in the world [7] but also in Sub-Saharan Africa (SSA) especially in children, women, and the elderly [4–7]. The majority of people in SSA, including rural residents and workers in the informal sector, have never had access to social or privately-run health insurance based on wages. Consequently, community-based health insurance (CBHI) schemes for urban and rural self-employed and informal sector workers have recently emerged as a response to the lack of social security, the unfavorable effects of user fees enacted in the 1980s, and the enduring issues with health care financing. That is, a promising effort to increase access to healthcare, health outcomes, and social protection in the event of illness appears to be CBHI. The CBHI approach may be especially beneficial because it allows for adaptation to local conditions, which is important given the distinctive ethnic, lingual, and cultural diversity within African nations, particularly Ethiopia [8].

Though various constraints have been hampering to address universal healthcare coverage (UHC) in Ethiopia, of all, financial hardship is the core. The catastrophic out of pocket (OOP) expenditure [9, 10] continues to be the main alternative of financing health care in Ethiopia [10] which was exceeded by 17.67% from the aggregated expense of SSA [11]. OOP expense is especially highest on medicines at poor households thereby hampering access to health care particularly if the health institutions are very far which led to indirect costs [12]. Thus, the Ethiopian government has been devoted to finding a way to shift from catastrophic OOP expenditure [13, 14] to ensure accessibility [11, 14] targeting quality and equity [15, 16] to achieve UHC [11, 17] particularly to increase the number of autonomous health institutions [12] and introducing the fee waiver program to protect the poor against untoward consequences of OOP payments [15]. As a result, health insurance has been taken as a strategy by the Federal Ministry of Health (FMOH) in 2008 [16, 18] with two schemes (risk-pooling arrangements) called social health insurance (SHI) for the formal sector and CBHI for the informal sector [12, 17, 19] to cover all citizens except defence forces [17].

The CBHI has been implemented since 2011 as a strategy for the road to UHC [5], which is the main target of Sustainable Development Goal (SDG) 3, to enable all people access to quality health services without financial hardship. Working on PHC is the most important strategy toward achieving UHC. However, the feasibility and effectiveness of this approach in low-income countries such as Ethiopia remain a source of concern [20]. Since the mid-1978, Ethiopia has placed PHC at the heart of its health-care system [3]. Nonetheless, insufficient service coverage, inequity of access, slow transition of health systems to provide services, poor quality of care, and high OOP expenditure remain major challenges, which may lead to beneficiary dissatisfaction [20]. In fact, responsiveness and accountability to beneficiaries are usually undervalued by providers in financing mechanisms [21]. However, CBHI is rooted in the self-decision and satisfaction of the households and could be affected by social, economic, and knowledge factors of the decision-maker (households’ head) [22].

**Fig. 1:**
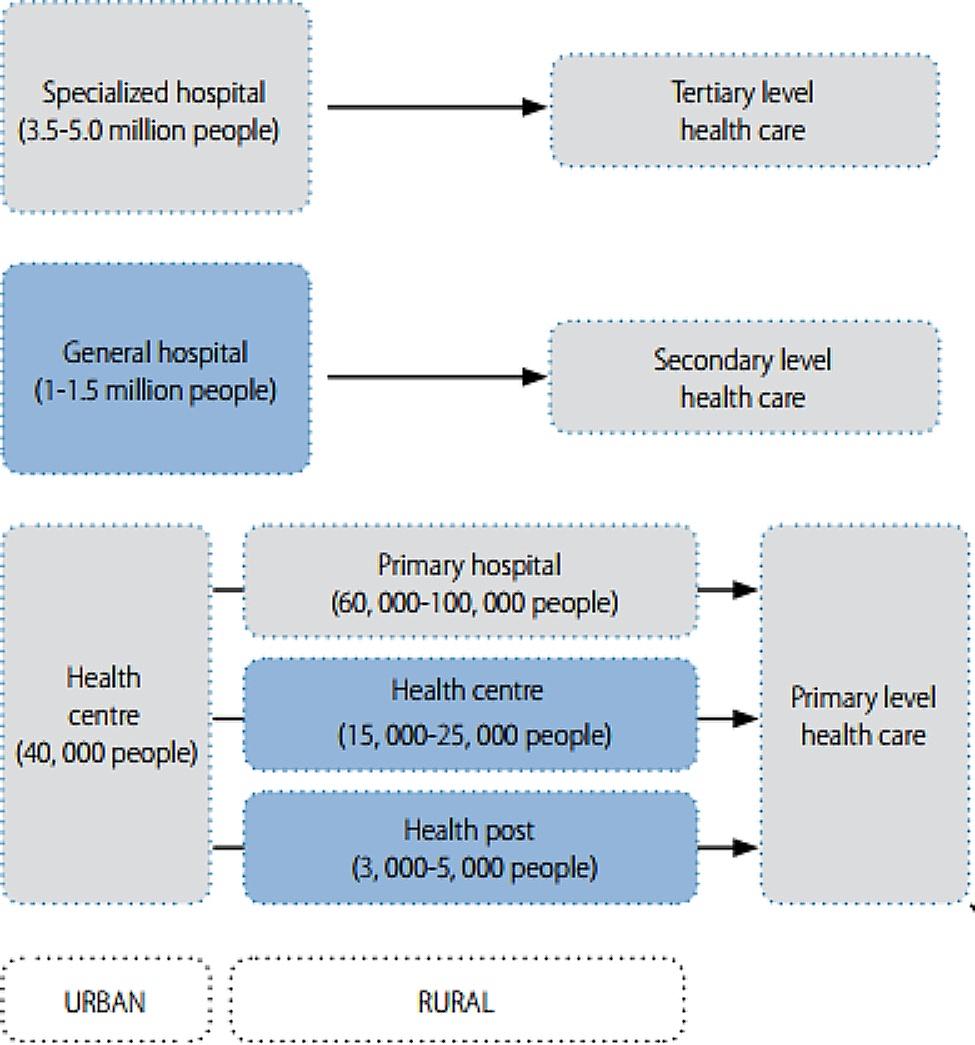
The health system organization (tire system) of Ethiopia [26]

**Fig. 2:**
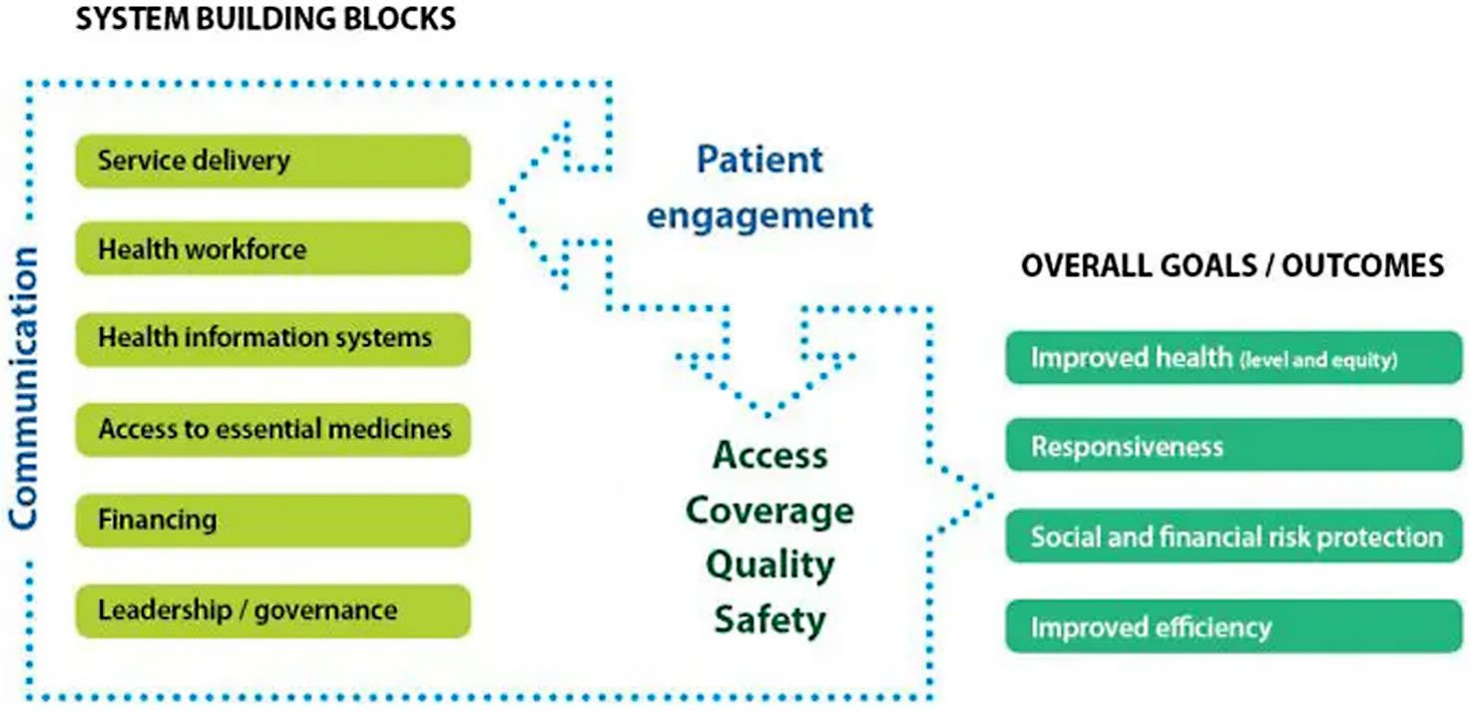
The modified WHO health systems framework [24]

Beneficiaries’ satisfaction is the beneficiaries’ evaluation of the quality of healthcare services provided by a health system [23], which consists of all the organizations, institutions, resources, and people whose primary purpose is to improve health [24]. For instance, the health service of Ethiopia is structured into a three-tier system: primary, secondary, and tertiary, which are interconnected to each other and for which the resort or referral system is from bottom to top (primary to tertiary) [25, 26] (Fig. 1). At each level of care, a health system has a responsibility to respond to beneficiaries’ expectations [23]. That is because without patient engagement (beneficiary satisfaction), the goal of a health system could never be achieved [24] (Fig. 2).

Though there is no a single standardized tool to measure them, beneficiaries’ reports and ratings of their experiences are the main tools healthcare systems use to measure their overall performance. As a result, measuring patient satisfaction is an important part of evaluating service quality and the responsiveness of the healthcare system [23], because the populations they serve—individuals, families, and communities—are the cornerstones of high-quality health systems. People are crucial partners in the delivery of healthcare and the improvement of health outcomes; they are not only the core beneficiaries of the healthcare system but also its agents, able to hold it accountable [27]. That means, beneficiaries’ satisfaction with services measures the degree of contentment with services received in a health system and serves as a proxy for the quality of healthcare [28]. However, despite more than 12 years of implementation of healthcare services with CBHI in Ethiopia as a risk-pooling mechanism, according to our information, there was no national pooled data to measure beneficiaries’ satisfaction. Therefore, this review aimed to evaluate the level of beneficiaries’ satisfaction with CBHI services and associated factors in Ethiopia. Accordingly, the main question to be answered by the review, using the CoCoPop Framework—Condition (satisfaction with CBHI services), Context (Ethiopia), and Population (CBHI beneficiaries)—was: What is the level of beneficiaries’ satisfaction with CBHI services in Ethiopia?

## Methods

### Protocol and registration

The " Preferred Reporting Items for Systematic Reviews and Meta-Analyses (PRISMA) 2020 statement: an updated guideline for reporting systematic reviews” serves as the framework for all sections of the review [29] (Additional File 1). However, for the sake of simplicity and clarity, we used the PRISMA 2009 flow diagram [30] to depict the screening process of the studies, while we adequately discussed the screening process in words in accordance with the PRISMA 2020 flow diagram. The protocol of this review has been registered with PROSPERO, which is available at https://www.crd.york.ac.uk/prospero/display_record.php?ID=CRD42022356350.

### Eligibility criteria

We included cross-sectional studies reported in English that were conducted since 2012 and looked at beneficiaries’ satisfaction with CBHI. The studies were also selected using the following parameters: response rate, sample size, year of the study, context (regions), population (study units), and outcome variables. Studies with a high risk of bias and incomplete data were excluded. Additionally, the unpublished copy has been eliminated if a study had identical reports in both its published and unpublished versions. Also, we replaced preprints with their published versions when available during the revision stage [31, 32]. A study was also deemed a duplicate if it appeared in more than one journal, and the most recent publication was chosen to be included in the review.

### Information sources and search strategy

Database searches were completed on September 1, 2022, using Scopus, Research4Life (Hinari), PubMed, Google Scholar, and Semantic Scholar. Manual searches were conducted on the Hinari and PubMed databases; the “Publish or Perish” database searching tool version 8 has been used to search Scopus, Google Scholar, and Semantic Scholar [33] (Additional File 2). We also searched registries like the Ethiopian Health Insurance Services (EHIS) and the general web for additional information. Text words and indexed terms such as “satisfaction,” “community-based health insurance,” “factors,” and “Ethiopia” were used to search the databases. Year of study, publication year, content type, discipline, and language are other filtering methods that have been used. The references to studies that fulfilled the inclusion requirements were looked up in order to find more pertinent studies.

### Selection and data collection process

Two reviewers, EMB and HNT, independently screened the included studies after duplicates and irrelevant studies had been removed using Zotero reference manager version 6. These two researchers have carefully scrutinized the study selection. The articles were first refined by their title and abstract, then by full-text revision by these authors, first independently and finally jointly, until agreement was reached. In cases where there have been disagreements, a third reviewer has been contacted to settle the issue. Then, we included all studies that met the eligibility requirements and had a low or medium risk of bias.

For the purpose of collecting and abstracting data, a data extraction spreadsheet has been used. The data was independently extracted by EMB and HNT, who then compared their findings and came to an agreement. Otherwise, a third (guest) reviewer has been asked to review with these two reviewers in order to come to a consensus. Additionally, in order to access missing data, the study authors have been contacted.

The Excel spreadsheet has been used to extract the outcome variable, population (study units), year of study, context, sample size, response rate, and funding sources. The primary outcome of the review was satisfaction with CBHI. The factors influencing the level of satisfaction were additional outcomes.

### Study risk of bias assessment

Two reviewers, EMB and HNT, independently appraised the risk of bias for the included studies using the Joanna Briggs Institute’s tools (JBI). The following factors were used to evaluate the bias: the selection process, the study’s subjects and context, the measurement’s validity and dependability, confounding and mitigation techniques, and the suitability of the outcome measure. Studies with a score of seven or higher were classified as low risk, those with a score of five to six as medium risk, and those with a score of four or less as high risk. Then, the low- and medium-risk studies were included in the review. Any discrepancies were resolved through discussion and, if necessary, the involvement of a third reviewer.

### Effect measures and synthesis methods

For each study, the proportion of satisfaction with CBHI was calculated. In addition, the odds ratios (ORs) were calculated for the summary effects of the factors affecting the satisfaction level. Due to its menu-based interface and versatility in computing both proportions and other effect estimates, such as ORs, we used Stata 17 to compute the effect sizes, including the proportion of satisfaction and the ORs of the factors affecting it. Since the heterogeneity of the studies was above 50%, we used the random effects model. To compare the effect estimates across studies based on region (context), sub-group analysis was carried out. A p-value of less than 0.05 and a 95% confidence interval (CI) were used to calculate the overall statistical significance level.

### Reporting bias and certainty assessment

Between-study heterogeneity was assessed using the I^2^-statistic. Inverse variance (percentage of weight) was also used to calculate each study’s impact on the meta-analysis as a whole. The likelihood of publication bias among studies was examined using the Doi plot with an objective Luis Furuya-Kanamori (LFK) index. We also employed sensitivity analysis using fixed and random effects models to identify the source of heterogeneity or asymmetry.

## Results

### Study selection

**Fig. 3:**
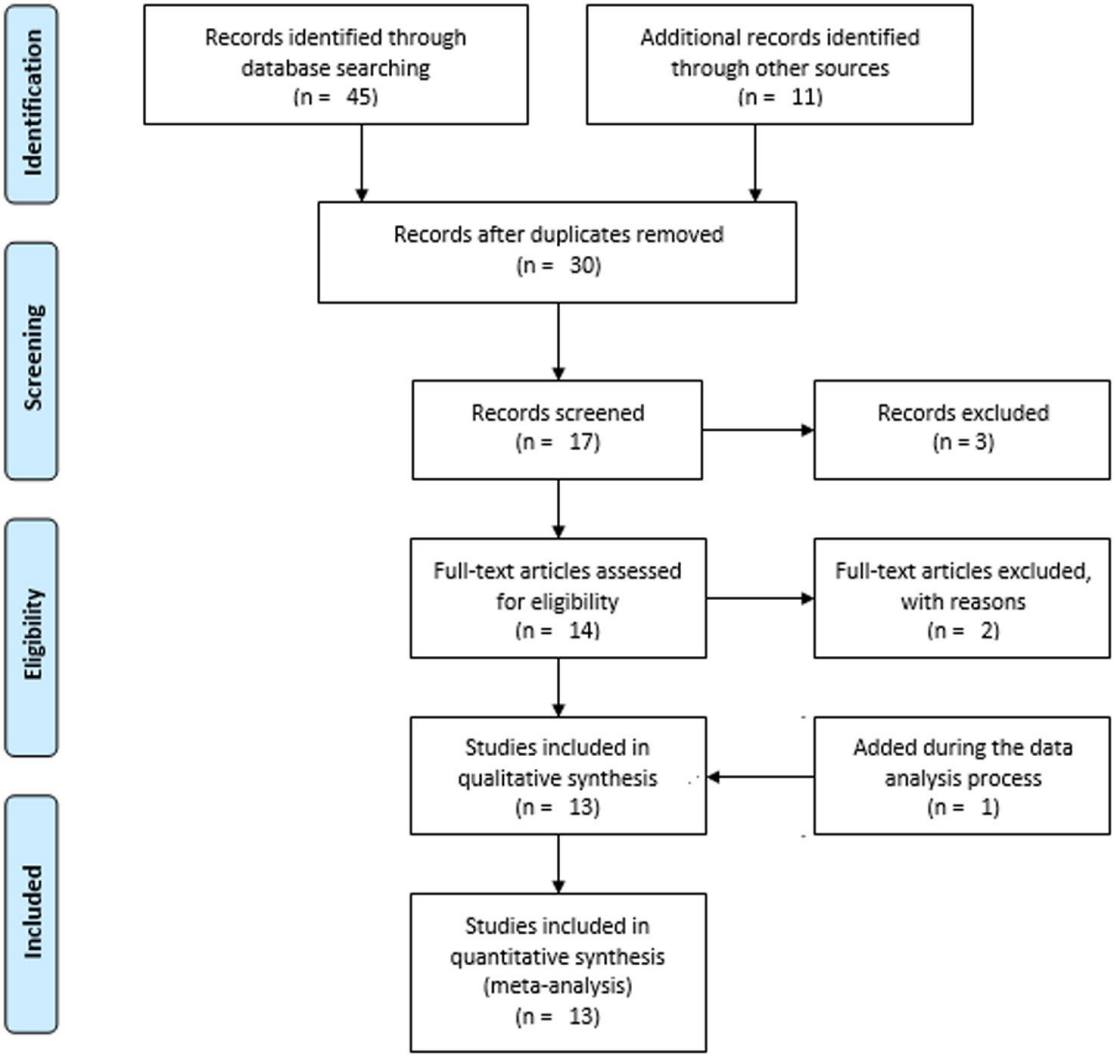
PRISMA flow diagram showing the selection processes of the included studies

There was a total of 56 resources found (Fig. 3). Databases, such as Scopus (*n* = 4), Hinari (*n* = 12), PubMed (*n* = 8), Google Scholar (*n* = 8), and Semantic Scholar (*n* = 13) were used to identify 45 of them. The remaining 11 sources came from websites (*n* = 9), and repositories (*n* = 2). After removing duplicates, 30 records were screened. Following 13 studies being removed due to a lack of relevance, 17 records underwent a title and abstract review. Using the titles and abstracts, 14 records were selected as deserving of full text analysis. Through the full text article evaluation, two articles were removed. Later, one article was discovered via web search and added during the data analysis process [34]. Finally, 13 studies were included for the systematic review and meta-analysis.

### Study characteristics

The majority of the studies that made up the systematic review and meta-analysis were carried out in the Amhara region (*n* = 5), followed by the SNNPR (*n* = 3) and Addis Ababa (*n* = 3). Study design, area (context), year of study, sample size, non-response and response rates, and primary outcome were all evaluated for each individual study (Table 1).

**Table 1:** Characteristics of the individual included studies, Ethiopia (*n* = 13), 2022

Study	Design	Area	Year	Outcome	SS	RR	Event	Quality
Fufa et al. 2021 [36]	Cross-sectional	Oromia	2019	Satisfaction	399	379	240	8/8
Hailie et al. 2021 [35]	Cross-sectional	Amhara	2019	Satisfaction	420	420	336	8/8
Badacho et al. 2016 [45]	Cross-sectional	SNNPR	2014	Satisfaction	386	386	353	5/8
Kebede et al. 2019 [43]	Cross-sectional	SNNPR	2018	Satisfaction	528	512	280	8/8
Gashaw 2020 [39]	Cross-sectional	Addis Ababa	2020	Satisfaction	366	366	194	5/8
Abera 2020 [31]	Cross-sectional	Amhara	2017	Satisfaction	311	311	247	5/8
Addise et al. 2021 [40]	Cross-sectional	SNNPR	2020	Satisfaction	627	562	304	7/8
Getaneh et al. 2019 [32]	Cross-sectional	Amhara	2019	Satisfaction	838	807	473	8/8
Yonas 2018 [41]	Cross-sectional	Amhara	2018	Satisfaction	399	377	271	5/8
Tefera et al. 2021 [38]	Cross-sectional	Nationwide	2019	Satisfaction	420	415	382	7/8
Balcha 2021 [42]	Mixed	Addis Ababa	2021	Satisfaction	419	404	187	5/8
Yasab 2021 [37]	Cross-sectional	Amhara	2021	Satisfaction	630	604	339	8/8
Haile et al. 2022 [34]	Cross-sectional	Addis Ababa	2021	Satisfaction	785	761	458	8/8
**Total**					6528	6304	4064	6.7/8

*Note* RR: Response Rate; SS: Sample Size

### Risk of bias in the included studies

**Fig. 4:**
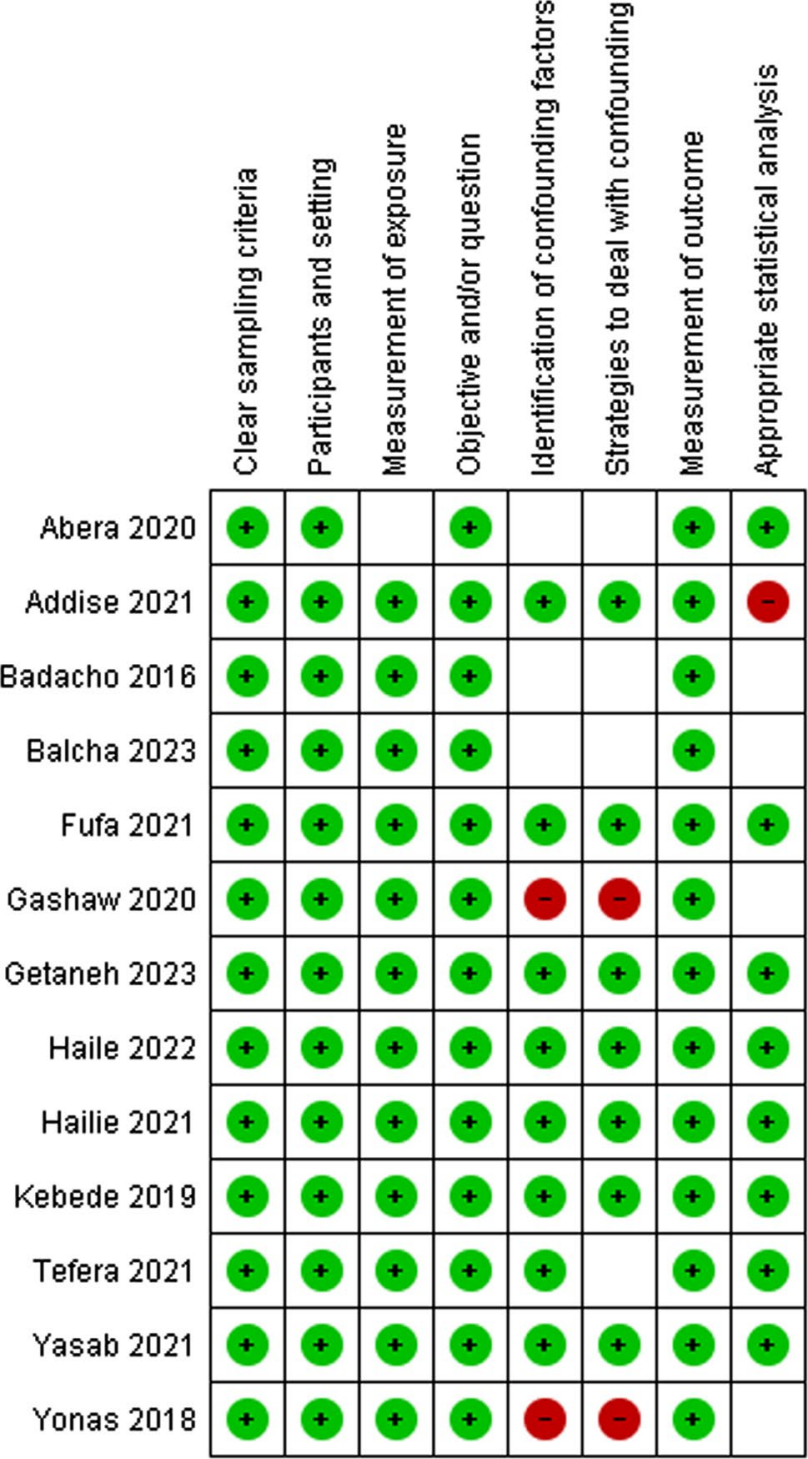
The risk of bias of the included studies (*n* = 13): Green = low risk, Red = high risk, Unfilled = unclear risk

The risk of bias for the included studies was assessed using an eight item JBI’s critical appraisal tool (Fig. 4). Then, those studies with low and medium risk were included in the review. As shown in Table 1, the average risk of bias in the studies has been 6.7 (83.8%).

### Results of synthesis

#### Proportion of beneficiaries’ satisfaction

**Fig. 5:**
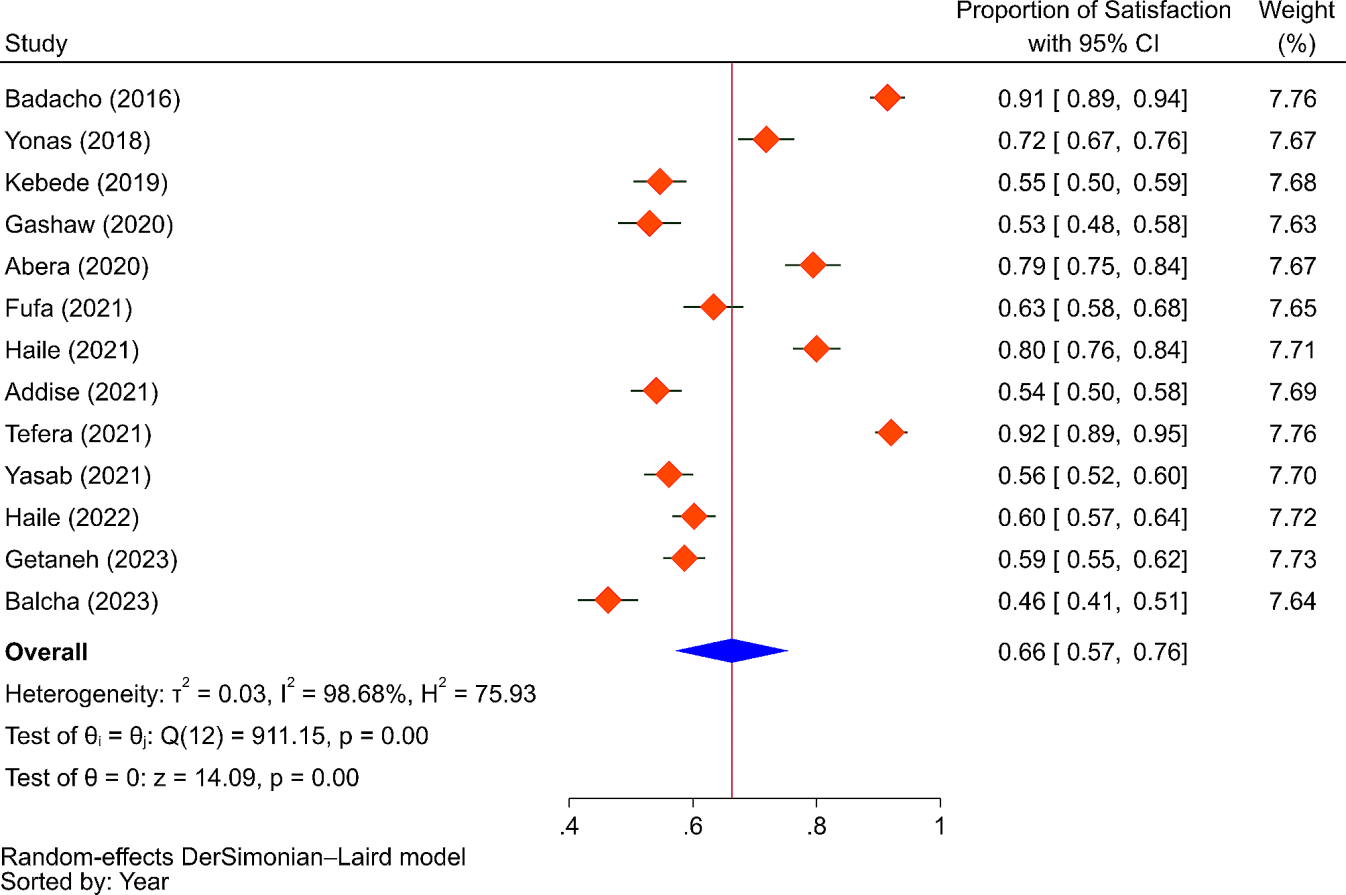
The pooled result of the proportion of beneficiaries’ satisfaction with CBHI

In total, 6528 household heads made up the sample population for all the included studies, and 6304 of them—or 96.6%—were actual participants. We have summarized the findings of each included study’s individual characteristics in Table 1. For the pooled analysis, 6,304 participants (the actual participants) were included from 13 studies, of which 38.6% were from the Amhara region, and the rest were from Addis Ababa (23.5%), SNNPR (22.4%), and Oromia (5.8%) regions, as well as nationwide studies (6.4%). The pooled satisfaction of beneficiaries with CBHI was found to be 66.0% (95% CI = 57-76%) (Fig. 5).

Table 2 illustrates a sub-group analysis by region, indicating that beneficiary satisfaction levels were highest in the Amhara region at 69.0% (95% CI = 59-79%), followed by SNNPR at 67.0% (95% CI = 40-94%), Oromia at 63.0% (95% CI = 58-68%), and Addis Ababa at 53.0% (95% CI = 45-62%).

**Table 2:** The sub-group analysis of beneficiaries’ satisfaction with CBHI in Ethiopia by region (*n* = 13), 2022

Region		Proportion (95% CI)	Weight (%)
**SNNPR**	Badacho et al. 2016 [45]	0.91 [0.88, 0.94]	7.76
	Kebede et al. 2019 [43]	0.55 [0.50, 0.59]	7.68
	Addise et al. 2021 [40]	0.54 [0.50, 0.58]	7.69
	**Subtotal**	**0.67 [0.40, 0.94]**	**23.13**
**Amhara**	Yonas 2018 [41]	0.72 [0.67, 0.76]	7.67
	Abera 2020 [31]	0.79 [0.75, 0.84]	7.67
	Hailie et al. 2021 [35]	0.80 [0.76, 0.84]	7.71
	Yasab 2021 [37]	0.56 [0.52, 0.60]	7.70
	Getaneh et al. 2019 [32]	0.59 [0.55, 0.62]	7.73
	**Subtotal**	**0.69 [0.59, 0.79]**	**38.47**
**Addis Ababa**	Gashaw 2020 [39]	0.53 [0.48, 0.58]	7.63
	Haile et al. 2022 [34]	0.60 [0.57, 0.64]	7.72
	Balcha 2021 [42]	0.46 [0.41, 0.51]	7.64
	**Subtotal**	**0.53 [0.45, 0.62]**	**23.00**
**Oromia**	Fufa et al. 2021 [36]	0.63 [0.58, 0.68]	7.65
**Nationwide**	Tefera et al. 2021 [38]	0.92 [0.89, 0.94]	7.76
**Overall**		**0.66 [0.57, 0.76]**	**100.00**

#### Factors affecting the beneficiaries’ satisfaction

The qualitative synthesis showed that the beneficiaries’ satisfaction with CBHI has been found to be affected by socio-demographic factors like age [31, 35–38], sex [31], education level [31, 36, 38, 39], income [40], occupation [31], marital status [31, 35], family size [34, 38], and residence [37]; health service related factors such as service quality [38, 41], confidence and friendliness with healthcare providers [34, 37, 42], waiting time [34–36, 39], laboratory services [32, 34, 38–40, 43], availability of medicines [34–38, 40, 42], immediate care [32, 42], referral service [32, 35, 36], and distance of health facilities [37]; the scheme’s related factors like regulation [42], affordability of premium [32, 36], office opening time [32], agreement with benefit packages [34] and time interval to use them [32], enrolment situation [32], and length of enrolment [43]; and knowledge regarding the scheme’s services [34, 37, 40, 43].

Among these listed factors, seven variables—knowledge regarding the scheme’s services, having prescription medicines, agreement towards the availability of laboratory services, residence, receiving immediate care, cleanliness of health facilities, and friendliness of health professionals—were found to be dichotomous. Consequently, they were entered into the meta-analysis regardless of their significance level in the original studies. Instead of taking the ORs reported, we used the proportion of participants provided in cross-tabulations from the original studies to calculate the pooled ORs.

**Fig. 6:**
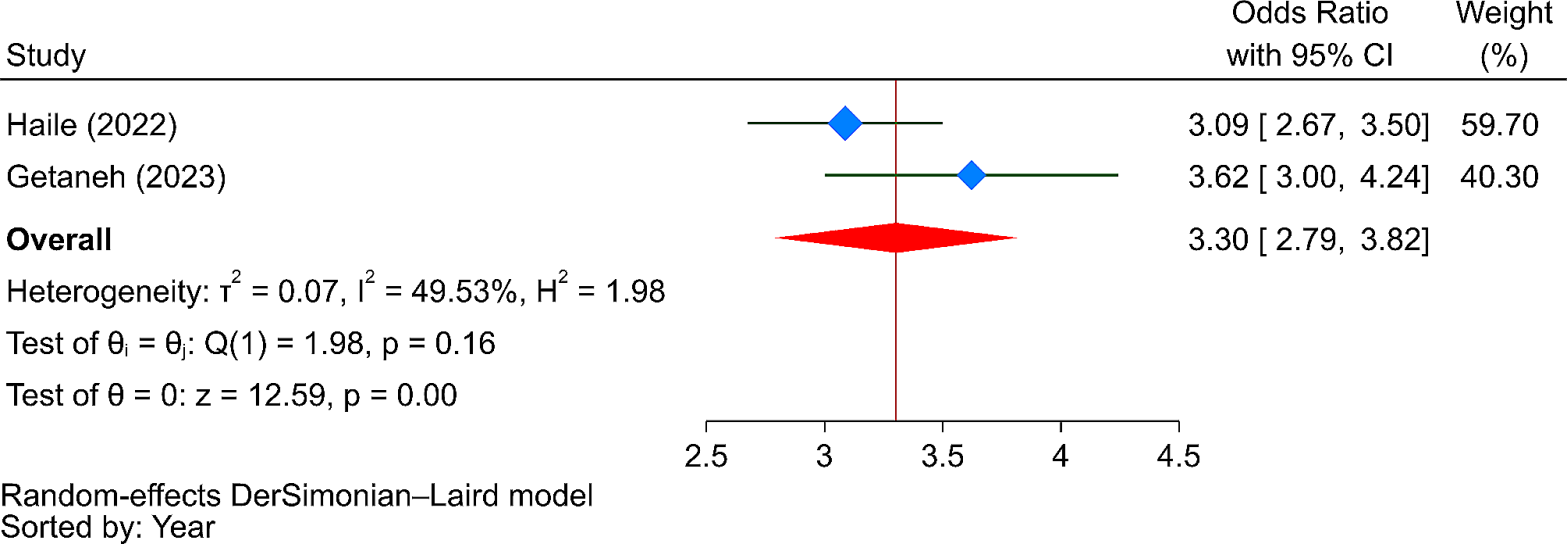
The strength of the relationship between the cleanliness of health facilities and satisfaction with CBHI services

Accordingly, participants living in urban areas were 2.32 times more likely to be satisfied with CBHI compared to their rural counterparts (OR = 2.32, 95% CI: 0.78–5.42). Regarding health service-related factors, those who received prescribed medicines (OR = 2.92, 95% CI: 0.04–5.81), had access to laboratory services (OR = 3.71, 95% CI: 0.15–7.26), received immediate care (OR = 2.15, 95% CI: -1.70-6.01), observed cleanliness of health facilities (OR = 3.30, 95% CI: 2.79–3.82), and perceived health professionals as friendly (OR = 5.14, 95% CI: -1.47-11.74) were more likely to be satisfied with CBHI services. Furthermore, individuals with good knowledge of CBHI services were 2.79 times more likely to be satisfied with CBHI services compared to those with poor knowledge (OR = 2.79, 95% CI: 0.43–5.16). However, as shown in Fig. 6, the only significant predictor of satisfaction with CBHI services was the cleanliness of health facilities.

#### Reporting bias and certainty of evidence

**Fig. 7:**
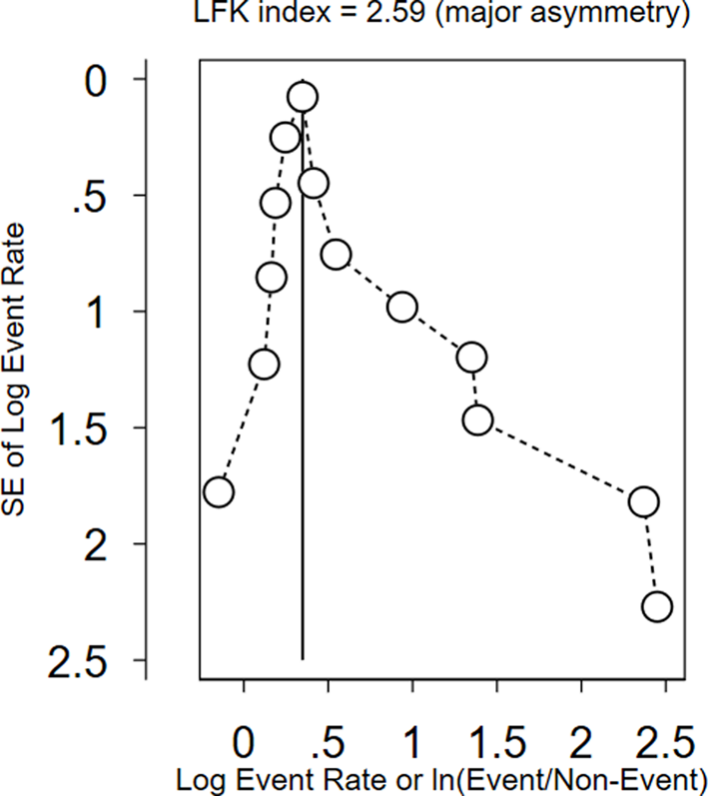
The Doi plot to assess publication bias between the included studies

**Fig. 8:**
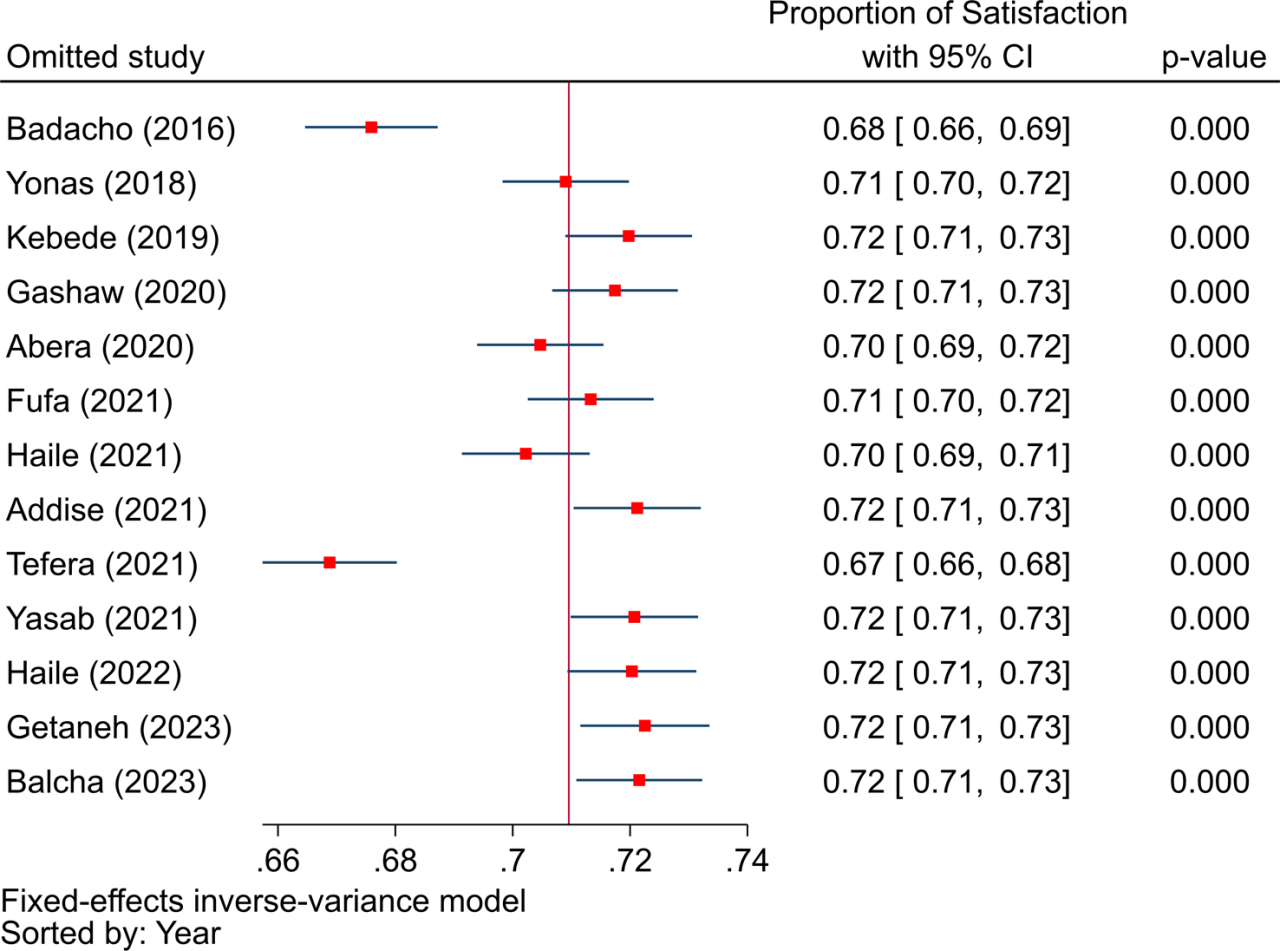
The sensitivity analysis to identify outlier studies from the included studies using the fixed effects model

**Fig. 9:**
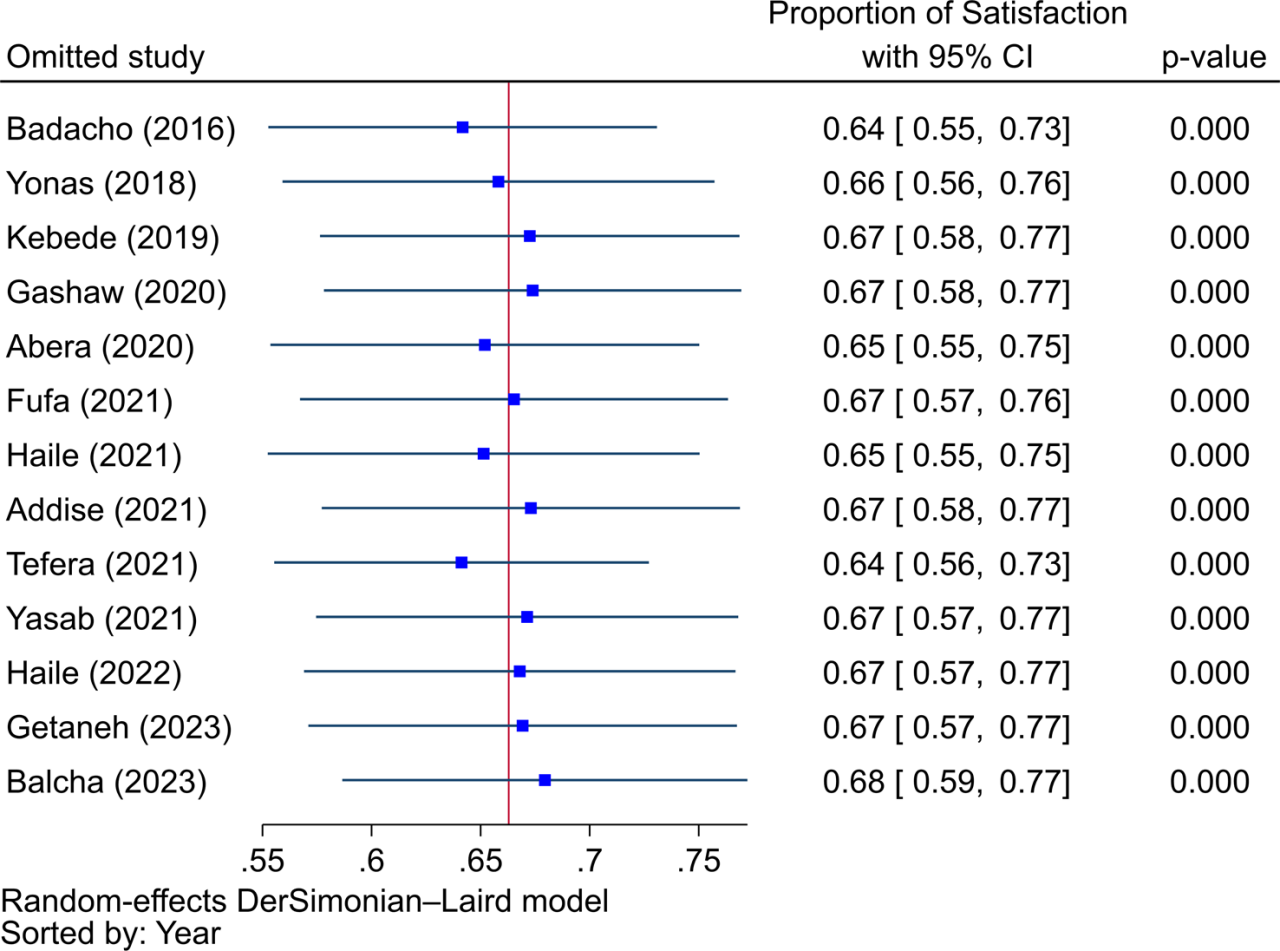
The sensitivity analysis to identify outlier studies from the included studies using the random effects model

In order to measure between-study heterogeneity, the I^2^ value was calculated. The sub-group analyses’ I^2^-values ranged from 0 to 99.37%, with an overall I^2^-value of 98.68%, which is an indicator of significant heterogeneity [44]. The Doi plot, as shown in Fig. 7, was used to investigate the possibility of bias among the included studies (publication bias), which yielded an LFK index of 2.59, indicating major asymmetry. Following this major asymmetry, we conducted sensitivity analysis to identify outlier studies, with five studies [32, 38, 40, 42, 45] found to be outliers with the fixed effects model (Fig. 8). However, when we employed the random effects model for the sensitivity analysis, none of the included studies were found to be outliers (Fig. 9). Therefore, a random-effects model was employed to pool the beneficiaries’ satisfaction with CBHI with a 95% CI [46].

## Discussion

This review revealed that the pooled satisfaction of beneficiaries with CBHI services was found to be 66.0%, which was quite higher than the satisfaction level of beneficiaries reported in other countries like Indonesia [47], Nigeria [48], and Saudi Arabia [49], at which the satisfaction levels were 34.76%, 42.1%, and 59%, respectively. Since the Government of Ethiopia (GOE) is working to strengthen the healthcare system to align it with the SDGs [50], the relatively higher level of satisfaction could be viewed as a blueprint for future efforts to achieve UHC and the SDGs by 2030. However, this does not necessarily imply that Ethiopian health-care quality is superior to that of those countries. Because the beneficiaries’ expectations in this nation might be low when compared to the expectations of beneficiaries in other nations. This is justified by the fact that when the expectations of beneficiaries are high, their satisfaction level with the scheme’s health services and the healthcare system as a whole drops, and vice versa; beneficiaries’ satisfaction is the gap between the expected and perceived characteristics of a service [51]. On the other hand, only 20% of the nation’s population had access to UHC services [52], which mandates a call for action to expand population coverage.

The beneficiaries’ satisfaction with CBHI services has been found to be affected by socio-demographic factors like age, sex, education level, income, occupation, marital status, family size, and residence; health service-related factors such as service quality, confidence and friendliness with healthcare providers, waiting time, laboratory services, availability of medicines, immediate care, referral services, and distance of health facilities; the scheme’s related factors like regulation, affordability of premiums, office opening times, agreement with benefit packages, enrolment situation, and length of enrolment; and the knowledge of households’ heads. These factors affect not only satisfaction but also the uptake of the scheme [53].

Among the most significant influencers of the need and demand for medical care [54], as well as satisfaction [55], are beneficiaries’ demographic and social factors. Ethnicity, gender, education level, health status [55], age [55, 56], family size, annual income [56], and marital status [48] are all known to affect how satisfied people are with their health care [56]. These factors might be crucial because the success and acceptance of CBHI programs depend greatly on community involvement, socioeconomic conditions, and cultural contexts [8]. Community participation will also improve how well the plan is understood and how well membership dues are paid. People’s overall satisfaction with the CBHI scheme’s services is therefore likely to increase when the scheme administrators have a tendency to pay attention to community preferences [57].

Though, in the meta-analysis, the only factor that was found to significantly influence it was the cleanliness of health facilities, the beneficiaries’ satisfaction with CBHI services is also significantly influenced by variables related to health care services. Likewise, other studies reported that the beneficiaries’ satisfaction was found to be influenced by the following health service factors: quality of service [58, 59], referral service [55], time spent during a visit (waiting time) [49, 55], availability of resources (doctors and medicines) [60, 61], access to care [49, 61], financial aspects of care (medical cost per family) [56, 61], diagnostic services, explanation about the prescribed medicine, the behavior of health personnel toward clients [62], the surrounding or waiting room environment of healthcare facility [58, 62], and the recovery by the patient [60]. The trust put into the process of providing services, however, was found to be far behind [63]. Because there is evidence that non-insured patients receive consultation, physical examination, and diagnosis services much more frequently than insured patients [31]. Due to this issue, beneficiaries hold the belief that seeking treatment at private medical facilities when seriously ill is preferable [63]. If appropriately regulated, this could be viewed positively. Because, in light of the fact that CBHI can complement other sources of funding rather than serve as a replacement for them, public-private partnerships may offer opportunities for improving CBHI performance [8]. However, all facilities in Ethiopia provide poor PHC, with scores ranging from 18 to 56% and a mean of 38% [64]. The beneficiaries did not feel that the service quality was satisfactory [41]. Concurrent issues include frequent drug shortages that cause frequent stockouts and lengthen the reimbursement process; high patient volumes that cause overcrowding in public health facilities; unnecessary price increases by private pharmacies for insurance beneficiaries; and uncertainty on annual renewal payments for services that are not used [63].

The other important factors influencing beneficiaries’ use of CBHI services were the following: length and duration of employment [48], comprehensiveness of covered health services (benefit packages) [49, 65], card processing time [66], ability to use health insurance to reduce medical costs via the co-payment mechanism [65], and general knowledge and awareness [48]. However, there is evidence that some important services were refused [49]. As a result, since patient satisfaction depends on the depth of insurance coverage and the ability to use health insurance to reduce medical costs via the co-payment mechanism, the inclusiveness of the benefit packages could be seen as a critical issue [65]. Moreover, beneficiaries’ awareness of health insurance is still limited [49].

### Limitations

Due to the different scales and doubtful calculations encountered in the original studies, we did not use the proportions and ORs provided by the authors for the meta-analysis to calculate the pooled estimates (proportions and ORs). Instead, we used the number of participants provided by the authors with cross-tabulations against the level of satisfaction, regardless of their significance level.

### Policy and practical implications

The SDGs reaffirm a global commitment to achieve UHC by 2030; all people and communities, everywhere in the world, should have access to the high-quality health services they need without facing financial hardship [67]. Healthcare quality, which includes people-centeredness, timeliness, equity, integration, efficiency, effectiveness, and safety, is mainly measured using beneficiaries’ satisfaction [67]. So, determining beneficiaries’ expectations is possibly the most crucial issue for health systems to address [55]. Therefore, to achieve UHC, health systems should track and report on the factors that matter most to people, such as quality care, user satisfaction, health outcomes, and system trust [27]. This should not be challenging because quality can be built into the foundations of health care systems, regardless of how far along they are on the path to achieving UHC [67]. Building the foundations of quality health systems must therefore be at the forefront of thinking, planning, and policy-making [67], because improving the quality of care will require system-wide action [27], which may also be crucial to expanding population coverage by CBHI.

### Direction to future research

Satisfaction and responsiveness are used to describe how well health systems, or specific parts of them, are able to meet the expectations of the general public or a specific patient population subgroup. The WHO claims that responsiveness is restricted “to the legitimate expectations of the population for their interaction with the health system” [68]. Therefore, since beneficiary expectations are the most important aspect to address in order for the health system to be more efficient and effective, health systems should prioritize those expectations in their strategies. However, to our knowledge, there were no comprehensive studies investigating beneficiaries’ expectations in Ethiopia. Thus, for the health system of Ethiopia to be more responsive to beneficiaries, further research investigating beneficiaries’ expectations seems crucial.

## Conclusion

The beneficiaries were found to be moderately satisfied with CBHI services. This could serve as a guide and motivating tool for the actions needed to achieve UHC and the SDGs. However, other measurement methods may be sought to determine whether the health system is in a good position to achieve UHC. Because the efficiency and effectiveness of the health system may not only be measured by the satisfaction of beneficiaries, in order to achieve UHC, which is the SDG’s main objective, additional efforts must be made as well.

## Electronic supplementary material

Below is the link to the electronic supplementary material.

Supplementary Material 1

Supplementary Material 2

## Data Availability

The data that support the findings of this study are available within the article.
