# Supplementary material for: Beneficiaries’ satisfaction with community-based health insurance services and associated factors in Ethiopia: a systematic review and meta-analysis
Source: Cost Eff Resour Alloc. 2024 Oct 18;22:73. doi: 10.1186/s12962-024-00541-4 (PMC11487762; doi:10.1186/s12962-024-00541-4)
Supplement: Supplementary file 2 — Supplementary Material 2 [file 12962_2024_541_MOESM2_ESM.docx]

**Database search**

1. **PubMed (n = 8):**

Search: **(((Satisfaction) AND (Community based health insurance)) AND (Ethiopia)) AND (("2012/01/01"[Date - Create] : "2022/09/01"[Date - Create]))**

("personal satisfaction"[MeSH Terms] OR ("personal"[All Fields] AND "satisfaction"[All Fields]) OR "personal satisfaction"[All Fields] OR "satisfaction"[All Fields] OR "satisfactions"[All Fields] OR "satisfaction s"[All Fields]) AND ("community based health insurance"[MeSH Terms] OR ("community based"[All Fields] AND "health"[All Fields] AND "insurance"[All Fields]) OR "community based health insurance"[All Fields] OR ("community"[All Fields] AND "based"[All Fields] AND "health"[All Fields] AND "insurance"[All Fields]) OR "community based health insurance"[All Fields]) AND ("ethiopia"[MeSH Terms] OR "ethiopia"[All Fields] OR "ethiopia s"[All Fields]) AND 2012/01/01:2022/09/01[Date - Create]

**Translations**

**Satisfaction:** "personal satisfaction"[MeSH Terms] OR ("personal"[All Fields] AND "satisfaction"[All Fields]) OR "personal satisfaction"[All Fields] OR "satisfaction"[All Fields] OR "satisfactions"[All Fields] OR "satisfaction's"[All Fields]

**Community based health insurance:** "community-based health insurance"[MeSH Terms] OR ("community-based"[All Fields] AND "health"[All Fields] AND "insurance"[All Fields]) OR "community-based health insurance"[All Fields] OR ("community"[All Fields] AND "based"[All Fields] AND "health"[All Fields] AND "insurance"[All Fields]) OR "community based health insurance"[All Fields]

**Ethiopia:** "ethiopia"[MeSH Terms] OR "ethiopia"[All Fields] OR "ethiopia's"[All Fields]

1. **Research4Life (R4L) - Hinari (n = 12):**

**Search:** (Satisfaction) AND (Community based health insurance) AND (Ethiopia)

Selected by:

**Date of publication:** from 2012/1/1 - 2022/9/01

**Content Type:** Any type

**Discipline:** Any type

**Language:** English

**Limit to:** Items with full text online

1. **Google scholar (n = 8)** **– using “Perish or Publish” software:**

**Satisfaction community based health insurance Ethiopia [title]**

*Publish or Perish 8.4.4041.8250 (basic report)
WinPosix (x64) edition, running on WinPosix 10.0.19042 (x64)*

**Search terms**

**Title words:** Satisfaction community based health insurance Ethiopia
**Years:** all
**Other options:** include citations; include patents

**Data retrieval**

**Data source:** Google Scholar
**Search date:** 2022-09-01 13:32:41 +0300
**Cache date:** 2022-09-01 13:32:46 +0300
**Search result:** [0] No error

***Important:*** *This data source provides only abbreviated data. Any ellipses (... marks) shown in this report originate with the data source; they are NOT caused by subsequent processing in Publish or Perish.*

**Metrics**

**Reference date:** 2022-09-01 13:32:46 +0300
**Publication years:** 2016-2021
**Citation years:** 6 (2016-2022)
**Papers:** 8
**Citations:** 51
**Citations/year:** 8.50 (acc1=4, acc2=2, acc5=2, acc10=0, acc20=0)
**Citations/paper:** 6.38
**Authors/paper:** 3.00/3.5/4 (mean/median/mode)
**Age-weighted citation rate:** 13.17 (sqrt=3.63), 4.85/author
**Hirsch h-index:** 2 (a=12.75, m=0.33, 47 cites=92.2% coverage)
**Egghe g-index:** 7 (g/h=3.50, 51 cites=100.0% coverage)
**PoP hI,norm:** 2
**PoP hI,annual:** 0.33
**Fassin hA-index:** 2

**Results**

AS Badacho, K Tushune, Y Ejigu, TM Berheto (2016) **Household satisfaction with a community-based health insurance scheme in Ethiopia**. *BMC research notes*, Springer, doi:10.1186/s13104-016-2226-9, cited by 32 (5.33 per year)

K Mitiku Kebede, SM Geberetsadik (2019) **Household satisfaction with community-based health insurance scheme and associated factors in piloted Sheko district; Southwest Ethiopia**. *PloS one*, journals.plos.org, cited by 15 (5.00 per year)

MT Hailie, SL Hassen, ... (2021) **Client satisfaction on community based health insurance scheme and associated factors at Boru Meda Hospital, Northeast, Ethiopia: institutional based cross …**. *BMC Health …*, bmchealthservres.biomedcentral …, doi:10.1186/s12913-021-07223-4, cited by 1 (1.00 per year)

T Addise, T Alemayehu, N Assefa, ... (2021) **The Magnitude of Satisfaction and Associated Factors Among Household Heads Who Visited Health Facilities with Community-Based Health Insurance …**. *Risk Management and …*, ncbi.nlm.nih.gov, cited by 1 (1.00 per year)

G Fufa, T Ermeko, AY Mohammed, ... (2021) **… Poor Community Based Health Insurance Client Satisfaction Level with Public Health Care Services in Negele Arsi Woreda Health Centers, West Arsi Zone Ethiopia**. *Health Science Journal*, itmedicalteam.pl

AF Abera, AY Desale, MD Argaw, BF Desta, ... (2020) **Satisfaction with Primary Health Care Services between Insured and Non-insured patients under Community-Based Health Insurance Scheme: A Comparative …**. *Fam Med Med Sci …*, researchgate.net, cited by 1 (0.50 per year)

B Aragaw (2019) **ASSESSMENT OF COMMUNITY BASED HEALTH INSURANCE WOMEN PATIENTS'SATISFACTION AND ASSOCIATED FACTORS AT FELEGE HIWOT …**., cited by 1 (0.33 per year)

L Yasab (2021) **Household Heads Satisfaction With Community Based Health Insurance Scheme and Associated Factors Among Enrollees of Bibugn District, East Gojjam …**.

1. **Scopus (n =** **4) – using “perish or publish” software:**

**Satisfaction community based health insurance Ethiopia [title]**

*Publish or Perish 8.4.4041.8250 (basic report)
WinPosix (x64) edition, running on WinPosix 10.0.19042 (x64)*

**Search terms**

**Title words:** Satisfaction community based health insurance Ethiopia
**Years:** all

**Data retrieval**

**Data source:** Scopus
**Search date:** 2022-09-01 13:32:09 +0300
**Cache date:** 2022-09-01 13:32:11 +0300
**Search result:** [0] No error

***Important:*** *This data source returns only one author per article; this affects the calculation of per-author metrics.*

**Metrics**

**Reference date:** 2022-09-01 13:32:11 +0300
**Publication years:** 2016-2021
**Citation years:** 6 (2016-2022)
**Papers:** 4
**Citations:** 21
**Citations/year:** 3.50 (acc1=2, acc2=2, acc5=0, acc10=0, acc20=0)
**Citations/paper:** 5.25
**Authors/paper:** 1.00/1.0/1 (mean/median/mode)
**Age-weighted citation rate:** 4.67 (sqrt=2.16), 4.67/author
**Hirsch h-index:** 2 (a=5.25, m=0.33, 21 cites=100.0% coverage)
**Egghe g-index:** 4 (g/h=2.00, 21 cites=100.0% coverage)
**PoP hI,norm:** 2
**PoP hI,annual:** 0.33
**Fassin hA-index:** 2

**Results**

A. Badacho (2016) **Household satisfaction with a community-based health insurance scheme in Ethiopia**. *BMC Research Notes* 9(1), ISSN 1756-0500, doi:10.1186/s13104-016-2226-9, cited by 14 (2.33 per year)

K.M. Kebede (2019) **Household satisfaction with community-based health insurance scheme and associated factors in piloted Sheko district; Southwest Ethiopia**. *PLoS ONE* 14(5), ISSN 1932-6203, doi:10.1371/journal.pone.0216411, cited by 7 (2.33 per year)

M.T. Hailie (2021) **Client satisfaction on community based health insurance scheme and associated factors at Boru Meda Hospital, Northeast, Ethiopia: institutional based cross-sectional study**. *BMC Health Services Research* 21(1), ISSN 1472-6963, doi:10.1186/s12913-021-07223-4

T. Addise (2021) **The magnitude of satisfaction and associated factors among household heads who visited health facilities with community-based health insurance scheme in Anilemo district, Hadiya zone, southern Ethiopia**. *Risk Management and Healthcare Policy* 14, pp. 145-154, ISSN 1179-1594, doi:10.2147/RMHP.S290671

1. **Semantic Scholar (n = 13) – using “perish or publish” software:**

**Satisfaction with community based health insurance in Ethiopia**

*Publish or Perish 8.4.4041.8250 (basic report)
WinPosix (x64) edition, running on WinPosix 10.0.19042 (x64)*

**Search terms**

**Keywords:** Satisfaction with community based health insurance in Ethiopia

**Data retrieval**

**Data source:** Semantic Scholar
**Search date:** 2022-09-01 13:39:13 +0300
**Cache date:** 2022-09-01 13:39:14 +0300
**Search result:** [0] No error

***Important:*** *The Semantic Scholar API is still under development and only provides limited paper search options and limited result data.*

**Metrics**

**Reference date:** 2022-09-01 13:39:14 +0300
**Publication years:** 2016-2022
**Citation years:** 6 (2016-2022)
**Papers:** 13
**Citations:** 67
**Citations/year:** 11.17 (acc1=5, acc2=5, acc5=2, acc10=0, acc20=0)
**Citations/paper:** 5.15
**Authors/paper:** 3.85/3.0/4 (mean/median/mode)
**Age-weighted citation rate:** 21.25 (sqrt=4.61), 6.61/author
**Hirsch h-index:** 4 (a=4.19, m=0.67, 63 cites=94.0% coverage)
**Egghe g-index:** 8 (g/h=2.00, 67 cites=100.0% coverage)
**PoP hI,norm:** 3
**PoP hI,annual:** 0.50
**Fassin hA-index:** 3

**Results**

B. Tefera, M. A. Kibret, Y. Molla, G. Kassie, Aynalem Hailemichael, Tarekegn Abate, Hailu Zelelew, B. Desta, E. Futrell, Zewditu Kebede, G. Abelti, S. Routh, B. Feyisetan, A. Saad (2021) **The interaction of healthcare service quality and community-based health insurance in Ethiopia**., doi:10.1371/journal.pone.0256132, cited by 3 (3.00 per year)

Kindie Mitiku Kebede, Sharew Mulugeta Geberetsadik (2019) **Household satisfaction with community-based health insurance scheme and associated factors in piloted Sheko district; Southwest Ethiopia**., doi:10.1371/journal.pone.0216411, cited by 15 (5.00 per year)

Teketel Addise, T. Alemayehu, N. Assefa, Desta Erkalo (2021) **The Magnitude of Satisfaction and Associated Factors Among Household Heads Who Visited Health Facilities with Community-Based Health Insurance Scheme in Anilemo District, Hadiya Zone, Southern Ethiopia**., doi:10.2147/RMHP.S290671

Girum Fufa, Tilahun Ermeko, A. Mohammed, A. Lette (2021) **Assessing Factors Associated with Poor Community Based Health Insurance Client Satisfaction Level with Public Health Care Services in Negele Arsi Woreda Health Centers, West Arsi Zone Ethiopia**., doi:10.36648/1791-809X.21.15.839

Girum Fufa, Tilahun Ermeko Wanamo, A. Mohammed, Abate Lette Wodera (2021) **Assessing Factors Associated with Poor Community Based Health Insurance for Client Satisfaction Level with Public Health Care Services in Negele Arsi District Health Centers, West Arsi Zone Ethiopia**., doi:10.36648/1989-5216.21.13.21

Mulugeta Tasew Hailie, S. L. Hassen, M. M. Temesgen (2021) **Client satisfaction on community based health insurance scheme and associated factors at Boru Meda Hospital, Northeast, Ethiopia: institutional based cross-sectional study**., doi:10.1186/s12913-021-07223-4

Abera Af, Desale Ay, Argaw, Desta Bf, Tsegaye Zt, Mavundla Tr (2020) **Patient Satisfaction with Primary Health Care Services between Insured and Non-insured patients under Community-Based Health Insurance Scheme: A Comparative Cross-Sectional Facility Based Study in North East Ethiopia**., doi:10.35248/2327-4972.20.9.245, cited by 1 (0.50 per year)

Abebe sorsa Badacho, K. Tushune, Yohannes Ejigu, T. Berheto (2016) **Household satisfaction with a community-based health insurance scheme in Ethiopia**., doi:10.1186/s13104-016-2226-9, cited by 24 (4.00 per year)

Z. Shigute, A. Mebratie, R. Sparrow, G. Alemu, A. Bedi (2020) **The Effect of Ethiopia’s Community-Based Health Insurance Scheme on Revenues and Quality of Care**., doi:10.3390/ijerph17228558, cited by 11 (5.50 per year)

M. Hussien, M. Azage, N. B. Bayou (2021) **Perceived Quality of Care in Health Centers Affiliated with a Community-Based Health Insurance Scheme in Two Districts of Northeastern Ethiopia: A Multilevel Analysis**., doi:10.1101/2021.10.18.21265144

Wakuma Akafu Eseta, Shimeles Ololo Sinkie (2022) **Factors affecting households’ trust in the community based health insurance scheme in Ethiopia**., doi:10.1371/journal.pgph.0000375

Teklemichael Gebru, Kifle Lentiro (2018) **The impact of community-based health insurance on health-related quality of life and associated factors in Ethiopia: a comparative cross-sectional study**., doi:10.1186/s12955-018-0946-3, cited by 13 (3.25 per year)

Dawit Gashaw (2020) **Level of Household Satisfaction in Community Baesd Health Insurance Beneficiaries on Medical Laboratory Services and Its Associated Factors in Selected Health Centers**., doi:10.7176/fsqm/98-04
